# Greek Physicians’ Skills and Factors Affecting Breaking Bad News to Cancer Patients

**DOI:** 10.3390/curroncol33050262

**Published:** 2026-04-30

**Authors:** Georgios Goumas, Theodoros I. Dardavesis, Konstantinos Syrigos, Nikolaos Syrigos, Dimitra S. Mouliou, Effie Simou

**Affiliations:** 1 Department of Public Health Policy, School of Public Health, University of West Attica, 11521 Athens, Greece; ggoumas@uniwa.gr (G.G.); esimou@uniwa.gr (E.S.); 2 Laboratory of Hygiene, Social & Preventive Medicine and Medical Statistics, School of Medicine, Faculty of Health Sciences, Aristotle University of Thessaloniki, 54124 Thessaloniki, Greece; dardaves@auth.gr; 3 Oncology Unit, 3rd Department of Medicine, “Sotiria” Hospital for Diseases of the Chest, National and Kapodistrian University of Athens, 15127 Athens, Greece; ksyrigos@med.uoa.gr (K.S.); nikolaos_syrigos@dfci.harvard.edu (N.S.); 4 Dana-Farber Cancer Institute, Boston, MA 02215, USA; 5 Independent Researcher, 38223 Volos, Greece

**Keywords:** breaking bad news, communication, skills, cancer, patients

## Abstract

Background/Objectives: Breaking bad news is crucial for patient-centered care. This study aimed to assess physicians’ skills and investigate the possible factors affecting their ability to communicate bad news. Methods: This web-based cross-sectional survey of 633 Greek physicians included demographic and other breaking bad news related questions to evaluate their skills and practices in breaking bad news to patients with cancer. Results: Most physicians defined bad news (91.5%) and frequently used both verbal and non-verbal communication (82.6%). About three-quarters rated their ability to communicate bad news as good to very good (75%) and about half (50.4%) disclosed bad news in a private and comfortable setting. Emotional responses, like sadness (53.4%) and compassion (49.6%), were common, while fears mainly focused on diminishing patients’ hope (60.8%) or managing patients’ reactions (53.9%). Female physicians showed higher anxiety (15.9% vs. 4.3%, *p* < 0.0005) and sadness (53.4% vs. 43.5%, *p* = 0.018) and lower self-perceived competence (*p* = 0.001) compared to males. Specialists and physicians with formal training demonstrated greater competence (*p* < 0.0005) and were more likely to choose private and comfortable settings (*p* < 0.0005). Multivariable logistic regression identified increased age (OR = 1.03; *p* = 0.018), male sex (OR = 1.63; *p* = 0.015), formal training in breaking bad news (OR = 9.34; *p* < 0.0005), residence outside Athens (OR = 2.27; *p* = 0.002), working in oncology (OR = 1.90; *p* = 0.043), and employment in private hospitals (OR = 1.81; *p* = 0.014) as statistically significant predictors of good to very good ability to communicate bad news to cancer patients. Conclusions: These findings highlight the value of structured training, targeted practice and institutional support in fostering physicians’ communication skills and boosting patient-centered care.

## Simple Summary

Explaining critical medical information to patients—particularly bad news—is a crucial part of good healthcare. This study examined how well Greek physicians communicate bad news and what factors influence their skills. In total, 633 physicians participated in an online survey on their experiences and feelings when breaking bad news. The majority of them were confident in their ability and used both dialogue and body language to communicate, while half of them ensured conversations occurred in private and comfortable settings. Moreover, several disclosed feeling sadness and compassion, whereas worries about taking away patients’ hope or handling emotional reactions were also reported. In addition, female physicians reported feeling more anxious and less confident than male physicians. Those with more experience, specialized training, or those working in certain settings (like private or oncology hospitals) disclosed some better communication skills. All in all, the research highlights that suitable training and support can aid in efficient communication of difficult information, care and support for patients and their families as well.

## 1. Introduction

In the 21st century, patient–doctor interaction has evolved significantly, shifting from paternalism to patient-centered care and shared decision-making. Engaged patients advocate for their health, challenging the traditional roles of physicians. This change affects healthcare professionals’ autonomy, highlights the Internet’s role in providing medical information, and impacts public health and health inequities [1]. Doctor–patient communication is not merely the exchange of words, but the meeting of two minds seeking to understand the human condition in its most vulnerable form. Therefore, it is evident that it is the most critical ingredient in medical care [2]. As AI increasingly affects healthcare communication, strengthening physicians’ communication skills is pivotal in maintaining human connection that boosts trust and empathy, especially in conditions such as cancer [3,4].

“Bad news” is defined as “any news that has a bad and severe effect on individuals’ perceptions of their future” and, in medical practice, its reporting is an inevitable and crucial responsibility of healthcare professionals [5]. Heretofore, the oncology and palliative care literature has progressively shifted from the term “breaking bad news” toward broader concepts like “serious illness communication” and “advance care planning,” reflecting an ongoing, patient-centered approach to communication across the trajectory of the medical condition. However, the term “breaking bad news” is still widely used in clinical practice and studies, especially when referring to the initial disclosure of a serious medical condition [6]. Even minor variations in how bad news is communicated can notably affect patients’ and families’ comprehension, attitudes, perceptions and treatment of the disease, underscoring the importance of effective communication strategies [5]. Physicians, particularly specialists facing high-stakes and challenging situations, such as oncologists, usually encounter critical challenges when disclosing cancer diagnoses, as patients and their families expect honesty, compassion, and support to help them deal with the condition [7]. Some related structured protocols provide a systematic approach to foster communication; nevertheless, gaps in training and psychological stress among healthcare providers continue to trammel the effectiveness of serious illness discussion [5,7]. Several structured communication training programs have been created to support physicians in delivering critical illness discussions and advance care planning. Despite the fact that the Setting, Perception, Invitation, Knowledge, Emotions, and Strategy (SPIKES) protocol is a widely used instrument in oncology for breaking bad news, palliative care education has expanded to incorporate broader frameworks like the Educating Physicians in End-of-Life Care (EPEC) curriculum and the VitalTalk program that emphasize ongoing, patient-centered communication of serious illnesses and goals-of-care discussions [8].

Cancer is characterized by uncontrolled growth of altered cells that undergo evolutionary changes influenced by natural selection, leading to the development of tumors and other complications and systemic inflammation may act as a predictor [9,10,11]. According to the World Health Organization (WHO), cancer is the second leading cause of death worldwide, accounting for nearly 10 million deaths in 2020, with approximately one-third of deaths linked to modifiable daily routine risk factors [12]. Globally, approximately one in five individuals will develop cancer during their lifetime, and the annual number of new cases is projected to increase to 35 million by 2050, representing a 77% increase compared with 2022 [12,13]. In 2022, although cancer incidence was the highest in Oceania, North America, and Europe, Greece showed notably high incidence and mortality rates, with cancer ranking as the third leading cause of death at the national level [14,15,16]. Not only is it crucial for cancer cases to have an immediate and prompt diagnosis and management of their condition but also to have excellent communication with healthcare professionals to combat the disease effectively.

The current literature reveals that notable studies have assessed healthcare professionals’ ability to communicate bad news [7,17,18,19,20]. However, to the best of our knowledge, thorough studies directly on patients with cancer are scarce. Particularly in Greece, where cancer rates are high compared to other countries, the relevant literature has mostly focused on the aspect of truth-telling, whereas there are only two studies—by Oikonomidou et al. [21] and Konstantis & Exiara [22]—based on some very small population-based samples, which try to investigate breaking bad news in clinical practice—yet the first one was a qualitative interview-based study and only the last one was solely targeted to patients with cancer and both of them have limited items. As a result, our research aims to further contribute to the present evidence by studying the communication of bad news in a large Greek population-based sample and by assessing possible independent predictors linked to physicians’ ability to communicate such information.

## 2. Materials and Methods

### 2.1. Study Design

The aim of this study was to investigate key aspects related to the communication of bad news of patients with cancer in healthcare settings and to reveal possible predictors for the physicians’ ability to deliver such information. Subsequently, a structured Web-Based Questionnaire (WBQ) was constructed, with items which were based on the previously published literature and guidelines towards the communication of bad news in clinical settings, especially the SPIKES protocol [21,22]. In order to boost content relevance and clarity, the final WBQ was reviewed by oncology and clinical communication experts, and minor adjustments were made to ensure its suitability for the Greek healthcare contexts.

The survey was carried out between June 2024 and August 2025. Greece has consistently faced health challenges and cancer remains a leading cause of death, with lung, breast, and colorectal cancers being the most prevalent. The country’s aging population, along with lifestyle factors such as smoking and obesity [22], contributes to high cancer rates. Nevertheless, heretofore, efforts to improve early detection and access to treatment seem to continue [23,24].

### 2.2. WBQ Design

This study was designed to explore key aspects of the communication of breaking bad news in healthcare settings and to identify factors associated with physicians’ ability and approach to delivering such information. To achieve this objective, a structured WBQ was developed based on the previously published literature and current guidelines addressing the communication of bad news in clinical settings, mainly the SPIKES framework. As a result, the items of the questionnaire were derived and adapted from the current literature on physicians breaking bad news, including Greek studies based on the SPIKES protocol, ensuring methodological consistency with previously used instruments and relevance to the local clinical contexts. In addition, similar questionnaire-based approaches investigating physicians’ attitudes toward delivering bad news have been applied in several previous studies without formal psychometric validation, since they primarily assess descriptive communication ways and professional perceptions and not latent psychometric constructs [17,20,21,22,25]. The WBQ was primarily based on binary and categorical items evaluating physicians’ self-reported practices, experiences, and perceptions towards breaking bad news. As a result, this study was designed in a descriptive and non-psychometric manner, and also it did not aim to assess latent constructs. Therefore, formal psychometric validation procedures (e.g., internal consistency reliability or factor analysis) were not applicable. Moreover, content validity was ensured via expert reviews by oncology and clinical communication specialists, who evaluated clarity, relevance, and appropriateness in Greek healthcare settings, and only few revisions were made based on their feedback. The WBQ was also informally piloted to ensure comprehensibility and feasibility before full distribution.

The first WBQs section consisted of information about the main purpose of the questionnaire and the study informing the potential participants that their personal data would be recorded anonymously and would be analyzed only for the purposes of this study by G.G., who would personally keep them. In addition, the classic closed-ended WBQs, which are based on qualitative categorical or dichotomous questions, are some familiar and advantageous psychometric attempts and seem to be desirable options for participation, in contrast to open-ended questions that require written answers. Consequently, the initial statement was followed by questions concerning demographic data, including binary questions on gender, working in Greece, completed specialty, as well as some categorical questions on family status, specialty category, and workplace, and a few open-ended questions on age, municipality of workplace, and the years completed after graduating from university and the years of work.

The subsequent WBQs section included items based on the SPIKES protocol and was compiled to investigate various aspects of physicians’ communication frameworks when delivering bad news. These included binary questions regarding whether physicians break bad news verbally and whether they choose to deliver such news while sitting near the bedridden patient [21,22,26]. Additionally, various categorical multiple-choice questions were included for the definition of breaking bad news, the preferable setting and manner by which such information is communicated, the truth’s disclosure and the individual’s involvement in the communication way. Some other questions tried to assess physicians’ reactions to patients’ statements and questions, the extent to which they investigate patients’ understanding during the communication process, and also their emotional experiences and possible fears when communicating bad news [21,22,26]. Further questions aimed to explore the individuals who support physicians as well as the ways they support them, along with the ways via which physicians reported having learned breaking bad news. Finally, a few items were included to estimate the frequency that the physicians communicate bad news, their self-perceived capability to communicate such information, and also their awareness of related communication protocols [21,22,26].

### 2.3. Population-Based Sample

A total of 633 physicians were finally included in this survey after the exclusion of 2 participants who reported to have an age of <18 years, 93 individuals who responded to only a few questions and 18 individuals who reported invalid answers. Therefore, a convenience and voluntary response sampling method was used in this study.

### 2.4. WBQ Administration

The questionnaire of this survey was shared through email and LinkedIn with physicians dealing with patients with cancer. Informed consent was obtained from all the participants during their participation in the study. WBQs were submitted to SurveyMonkey, and data were saved in an Excel sheet.

### 2.5. Statistical Analysis

Statistical analyses were performed using SPSS statistics for Windows, Version 31.0 (IBM Corporation, Somers, NY, USA). Missing data were few and were handled through listwise deletion, so analyses were solely performed on complete cases. Data normality was estimated using Kolmogorov–Smirnov test. The tests were two-tailed, and the level of statistical significance was set at *p* ≤ 0.05. Given that several variables deviated from normal distribution, mostly non-parametric tests were applied. Spearman coefficient was used to estimate correlations between variables, and Mann–Whitney U-test and Kruskal–Wallis H-test were used for estimates of mean ranks among subgroups. The chi-square test was applied for comparisons of frequencies, and Bonferroni correction was used for comparisons between subgroups to control for type I error because of the multiple comparisons. Correlations between categorical variables were assessed using the chi-squared test and Fisher’s exact test. A multivariable binary logistic regression model with the enter method was used to identify independent demographic predictors of the ability to communicate bad news (good to very good). ORs and 95% CIs are reported for all variables in the multivariable model. All the assumptions of the logistic regression analysis were examined (e.g., multicollinearity of independent variables and independence of error terms).

## 3. Results

### 3.1. Demographic and Other Information for the Population-Based Sample

The population-based sample of this study consisted of 633 physicians with a mean age of 47.1 ± 11 (25–82) years, of whom 400 (63.2%) were men. More than half of the participants (392; 61.9%) reported being married, whereas slightly below 3/4 (450; 71.1%) reported living in Athens.

The vast majority of the participants reported to be medical specialists (520; 82.1%) and, also, about 1/3 (217; 34.3%) reported to be surgeons, followed by internists (191; 30.1%), medical oncologists (133; 21%), radiologists (113; 17.9%) and others who disclosed other specialties. Moreover, about 1/4 (170; 26.9%) reported to be working in a university hospital and similar percentages were observed for general hospitals (161; 25.4%) and private hospitals (148; 23.4%), followed by those who reported to work in anticancer hospitals (75; 11.8%) and military hospitals (34; 5.4%). The mean duration of work experience as physicians was 18 (1–57) years.

### 3.2. Frequency, Methods, Self-Assessment and Place of Communicating Bad News

Table 1 illustrates information recorded from the participants about their belief in the definition of bad news, the frequency and way of announcing it to patients with cancer, their self-assessment of their ability to communicate bad news to patients with cancer, as well as the place they prefer to communicate with them.

### 3.3. Ways of Communicating Bad News to Cancer Patients

The majority of the participants (523; 82.6%) reported communicating bad news through verbal and non-verbal communication (touch, gaze, empathy, etc.), whereas the rest delivered it only via verbal communication. Moreover, most of them (450; 71.1%) reported that, in the case of a bedridden patient, they reported sitting near a patient’s bed, whereas the others preferred to stand by the bed. Table 2 shows further participant information on ways of communicating bad news to cancer patients.

### 3.4. Interaction and Support During the Communication of Bad News to Cancer Patients

Table 3 shows the participants’ information regarding interaction, feelings and fear during the communication of bad news to cancer patients.

In addition, about half of the participants (307; 48.5%) reported that they had learned how to communicate bad news by trying and learning from their own mistakes, and about 1/5 (127; 20.1%) disclosed learning during their studies. A similar percentage (122; 19.3%) was seen for those who reported learning during meetings with others, whereas the rest (72; 11.4%) reported having received formal training (formal training in breaking bad news was defined as any structured education or training program particularly targeting communication skills and the delivery of bad news to patients, as self-disclosed by participants).

Furthermore, the vast majority of the participants (570; 90.1%) disclosed the importance of incorporating breaking bad news as a course in the curriculum; however, only a few (48; 7.6%) reported being aware of any protocol relevant to breaking bad news to cancer patients.

### 3.5. Correlations and Further Analyses

Some further data processing revealed some correlations between variables. Table 4 reveals further correlations between a few variables related to breaking bad news and gender, hospital type, type of physicians, and their experience breaking bad news.

A multiple logistic regression model with the enter (simultaneous entry) method was applied to examine the correlation between demographic characteristics and the likelihood of reporting good to very good ability to break bad news. Increased age [OR(95%CI): 1.03(1–1.1); *p* = 0.018], male sex [OR(95%CI): 1.63(1.1–2.4); *p* = 0.015], training for breaking bad news [OR(95%CI): 9.34(2.9–30.6); *p* < 0.0005], physicians living outside Athens [OR(95%CI): 2.27(1.4–3.8); *p* = 0.002], working in oncology [OR(95%CI): 1.90(1–3.5); *p* = 0.043] or private hospital [OR(95%CI): 1.81(1.1–2.9); *p* = 0.014] were associated with good to very good ability to communicate bad news, as seen in Table 5.

## 4. Discussion

This Greek cross-sectional study included 633 physicians (mean age 47.1 ± 11 years; range 25–82) who coped with patients with cancer, most of whom were men (63.2%), married (61.9%), and residing in Athens (71.1%). The majority were medical specialists (82.1%), representing various specialties, with a mean duration of professional experience of 18 years (range 1–57 years), and were employed across divergent healthcare settings, including university, general, private, and anticancer hospitals. Most physicians (91.5%) defined bad news as information implying a negative change that has an impact on an individual’s life and future, and more than half (54.4%) communicated bad news daily or frequently. A large proportion (82.6%) used both verbal and non-verbal communication, while 75% believed they had a good to very good ability to break bad news; however, only about half (50.4%) did this in a private and comfortable setting and half of them preferred to report bad news first to the patient’s family. In their qualitative interview-based study, which was based on 25 resident and specialist doctors for patients in general, Oikonomidou et al. [21] found that doctors’ practices were mostly shaped by clinical experience rather than formal guidelines and used to be affected by family’s requests. Konstantis & Exiara [22] found also that residents engage less frequently in communicating bad news than specialists, revealing that professional experience greatly affects the communication practices. In another study of Ferreira Da Silveira et al. [26], this definition of bad news showed almost total agreement (99.17%), regarding the way in which communication rates were similar (84%), but most of them considered their ability to communicate as acceptable to good, and higher rates (78%) were reported for communication in a private and cozy place, and this study reported differences in age and years of work. Such differences may be realistic but were absent from our study. Moreover, in their study, Al-Mohaimeed and Sharaf [27] found that the majority of physicians communicate bad news only to the patient’s family but, in our study, doctors reported discussing the truth both with the patient and the family regardless of who will be the first to announce it.

Furthermore, in our study, clear, jargon-free language was used by 84.8% of participants, and 92.6% carefully tailored the information to patients’ and/or families’ needs, although fewer physicians provided thorough explanations (38.9%), expressed hope in the absence of favorable prospects (26.7%), or put themselves in the patient’s position (28.8%). Furthermore, most of them showed attentive communication behaviors, with 67.5% listening without interrupting and 72.0% always having time to answer patients’ questions, while about three quarters explored patients’ existent knowledge (79.0%), information preferences (75.0%), and concerns (79.5%). This aligns with the study of Oikonomidou et al. [21] that highlighted the value of an individualized, patient-centered way, advocating comprehensible information, recommendations, and empathy. On the other hand, Spiegel et al. [17] revealed that patients perceived family doctors as more empathic than hospital-based specialists and were more likely to feel they had enough opportunity to ask questions, highlighting the value of personalized, empathic communication and coordinated care for a better patient satisfaction during the communication of cancer and breaking bad news. Konstantis & Exiara [22] similarly found that Greek doctors prioritize private, comfortable settings and clear, easily understandable communication language and frameworks. Compared to our results, Ferreira Da Silveira et al. [26] reported similar rates for comprehensible language (85.95%) but fewer responded to patients’ questions (56.2%). Another study of healthcare specialists agreed with these results by revealing that doctors preferred non-technical and easy comprehensive language and used both verbal and non-verbal communication [28]. The findings of another study showed that physicians strongly preferred communicating bad news in person and in private, giving hope to patients, and assessing patients’ understanding of their condition, while less frequently engaging in physical gestures like placing a hand on the shoulder or via phone communication [7].

The most commonly reported emotions when breaking bad news were compassion and sadness (49.6%) and a sense of duty fulfillment (30.6%), while fears centered on reducing patients’ hopes (60.8%) and dealing with patients’ reactions (53.9%). Even if most of them provided informational (90.5%), practical (89.4%), and emotional support (85.2%) to both patients and their families, only half had learned how to communicate bad news via trial and error (48.5%) but (92.4%) were unaware of formal protocols for the communication of bad news. Oikonomidou et al. [21] similarly showed that doctors experienced emotional distress and fear concerning patients’ reactions, with disclosure practices mostly guided by observation of senior colleagues and intuition as well. Konstantis & Exiara [22] revealed that many physicians rely on experiential learning instead of formal education and several are unaware of communication guidelines; however, they acknowledge the importance of structured training to deal with both informational and emotional aspects of breaking bad news effectively. Despite our low percentage of participants who knew relevant protocols, none of the participants of the study of Ferreira Da Silveira et al. [26] knew such protocols, yet the sample size was very low [26]. Indeed, another study on physicians in general [25] showed that only a few of them had received relevant training. In addition, a study including both oncology healthcare specialists and medical students also highlighted the need for related training, such as seminars or conferences [29]. Training in communication skills and learning how to balance emotional involvement without fear are essential, emphasizing that specialized training greatly improves doctors’ ability to handle such sensitive situations compassionately and competently [30]. In addition, it has been discussed that qualities such as empathy and a sense of responsibility are critical for delivering bad news effectively [31].

In our study, female physicians tended to use highly emotional communication (*p* = 0.002) and lower self-perceived competence (*p* = 0.001) than men, but they experienced higher anxiety (15.9% vs. 4.3%, *p* < 0.0005), sadness (53.4% vs. 43.5%, *p* = 0.018), and fear levels related to both their personal emotional reactions (*p* = 0.011) and patients’ reactions (*p* = 0.004). In contrast, male physicians tended to have a more frequent sense of duty fulfillment (*p* < 0.0005) and no fear (*p* = 0.003), whereas no sex-related differences were observed in the workplace setting (*p* = 0.843) or training mode (*p* = 0.220). Interestingly, specialists showed notably higher competence (*p* < 0.0005) and were more likely to communicate bad news in a comfortable and private setting (*p* < 0.0005) than physicians in training. Differences in ability were also highlighted across hospital settings (*p* = 0.002), with physicians in public hospitals showing a more frequent poor to acceptable ability than those in private hospitals (*p* < 0.0005), with the latter more often selecting private and comfortable environments for breaking bad news (*p* < 0.0005). Moreover, physicians with formal training in breaking bad news tended to use emotional communication frequently (*p* < 0.0005), showed higher competence (*p* < 0.0005), and usually selected a comfortable and private setting (*p* < 0.0005) compared to those who acquired that ability empirically (*p* = 0.006). Finally, in our study, a multiple logistic regression model identified factors linked to good to very good ability to communicate bad news, and higher ability was attributed to increased age (OR = 1.03; *p* = 0.018), male sex (OR = 1.63; *p* = 0.015), formal training in breaking bad news (OR = 9.34; *p* < 0.0005), residence outside Athens (OR = 2.27; *p* = 0.002), working in oncology (OR = 1.90; *p* = 0.043) and private hospitals (OR = 1.81; *p* = 0.014). It is worth discussing that the observed gender differences in self-perceived competence and emotional response could have been affected by multifarious factors, such as differences in seniority and clinical experience, possible hierarchical structures in healthcare settings, and gender-relevant discrepancies in self-assessment and emotional reporting; particularly, male physicians are frequently more capable of occupying senior roles, which could contribute to higher confidence in communication tasks, whereas female physicians can have higher emotional engagement and self-critical assessment, but such interpretations must be considered exploratory and need further investigation. It should also be highlighted that the strong association seen between training in breaking bad news and perceived communication ability (OR = 9.34) must be interpreted with caution, since residual confounding cannot be excluded in this cross-sectional design; yet, this finding possibly reflects the substantial effect of structured communication training on physicians’ perceived competence. Moreover, it should be noted that differences between private and public hospital settings in Greece may show variations in patient socioeconomic status, caseload, and available time for doctor–patient communication, which could partially explain the observed linkages. Oikonomidou et al. [20] also highlighted that individual, interpersonal, and organizational factors—like emotional distress, relationships with patients, family involvement, time constraints, and poor space—have a notable impact on disclosure practices. In addition, Konstantis & Exiara [21] revealed that mostly physicians’ sex, experience, and former training determined confidence and competence in the communication of bad news, with specialists and previously trained doctors to report greater comfort, more structured communication ways, and more efficient management of emotional obstacles.

Our study had several important strengths. First, it was based on a large population-based sample of 633 physicians, thus enhancing the generalizability of the results. Second, the use of a WBQ allowed the collection of standardized data and facilitated answers from busy healthcare professionals, as well as both closed-ended and Likert-type questions, which ensure quantifiable and analyzable data and capture nuanced perceptions. Anonymity and informed consent were preserved; therefore, the reports were honest and ethically rigorous, whereas our strict exclusion and inclusion criteria ensured the validity of the dataset. Furthermore, our questionnaire was based on the validated SPIKES protocol, and it addressed various dimensions of bad news communication, such as ways of communication, emotional responses and ways of support. Even if the SPIKES protocol was used as the principal basis for our study, it is crucial to disclose that contemporary palliative care education has expanded beyond single-event bad news delivery frameworks, and several other programs such as EPEC and VitalTalk provide structured training in ongoing serious illness communication and advance care planning, revealing a wider and more longitudinal approach to communication across the trajectory of serious illness. In addition, the questionnaire included demographic, work-related, and practice-relevant questions, allowing multivariate analyses of factors that may have an impact on breaking bad news skills.

Nevertheless, several limitations should be considered, such as the web-based design that may have introduced some selection bias in favor of physicians who are more comfortable with the Internet and online tools. Because of the open and non-random nature of dissemination via professional networks and social media platforms, the precise number of physicians who received the invitation cannot be counted; therefore, a direct response rate cannot be calculated. This recruitment strategy triggers selection bias, since participation was voluntary and possibly more likely among doctors interested in communication in oncology; yet, it allowed access to a broad and geographically different sample of physicians in real-world practice settings. Secondly, the cross-sectional design precludes causal inferences among demographic or professional factors and communication ability, whereas some participants may have provided incomplete answers in a few open-ended questions, thus limiting qualitative insights. In addition, social desirability bias, especially concerning competence and emotional responses, may have affected the self-reported information. Additionally, some subgroups, such as physicians who work outside major urban centers like Athens, may be underrepresented. Furthermore, recall bias may have an impact on answers concerning past training or experiences with communicating bad news. It is also important to note that our study did not account for institutional or cultural variations in hospital policies that may affect practices towards serious illness discussion, whereas possible differences in patient populations among specialties were not controlled for, a fact that may influence the generalizability of our results. Finally, the absence of follow-up makes identification and comprehension of the ways in which communication skills improve over time more difficult.

Future directions include tracking the evolution of communication in general and particularly breaking bad news skills over time and after certain interventions. Furthermore, some randomized controlled trials of formal breaking bad news training programs may provide stronger evidence of direct causality. In addition, the inclusion of qualitative interviews or focusing on specific groups may provide valuable insights into the emotional and ethical obstacles that physicians may face. In addition, the generalizability and the representativeness of such results can be achieved by including physicians from various regions and smaller hospitals. Moreover, a better and more accurate estimation of the efficacy of breaking bad news may be provided by studying patients’ perspectives alongside (their) physician reports. It would also be of notable importance to study the influence of various cultural, institutional, and specialty-related factors on communication strategies, to reveal context-relevant best strategies. Another crucial strategy may be the integration of objective measures of skills, such as observed patient interactions or simulation-based evaluations, which can complement and strengthen self-reported information. In addition, assessment of the long-term results of the evolved communication on patient satisfaction, adherence, and clinical results can help associate training and practice with notable advantages. Finally, and related to our country, since there are currently no Greek national standardized guidelines particularly regulating the communication of bad news and physicians typically acquire relevant skills through medical education, specialty training, continuing professional development activities, and exposure to internationally recognized frameworks such as the SPIKES protocol, it would be worth having such guidelines for our physicians as well.

## 5. Conclusions

This study provides crucial insights into Greek physicians’ practices and obstacles in breaking bad news to patients with cancer. Despite the fact that most physicians showed adequate self-perceived competence and tended to use both verbal and non-verbal communication methods, there still exist gaps in the consistent usage of private and supportive settings and in dealing with the emotional burden attributed to this process. The observed differences relevant to sex, experience, and training highlight that communication competence in the communication of bad news is affected not only by clinical exposure but also by structured education and workplace settings. Such findings highlight the essence of systematic incorporation of communication education—especially structured protocols, recommendation frameworks and experiential learning—into under- and post-graduate medical training. Moreover, institutional policies which ensure suitable environments, protected time for the communication with patients and their families as well, and interdisciplinary support could boost physicians’ confidence and effectiveness in breaking bad news. Future research could further investigate patients’ views and assess the impact that targeted educational interventions have on both physician performance and patient results, which can further contribute to the evolution of culturally sensitive and patient-centered communication frameworks in oncology care.

## Figures and Tables

**Table 1:** Frequency, methods, self-assessment and place of communicating bad news to cancer patients.

		*N*	%
What is bad news?	Any information related to the presence of physical injury to the patient	39	6.2
	The news of death	15	2.4
	Any transmitted information that implies a negative change and could affect the person’s view of life or their prospects for the future	579	91.5
How often do you announce the bad news to patients with cancer?	Daily	82	13.0
	Very often	262	41.4
	Occasionally	173	27.3
	Seldom	109	17.2
	Never	7	1.1
How do you assess your ability to communicate the bad news to patients with cancer?	Very good	170	26.9
	Good	302	47.7
	Acceptable	149	23.5
	Bad	12	1.9
Which place do you choose to announce the bad news to patients with cancer?	Looking for a private and comfortable place	319	50.4
	I choose to update at an available office	298	47.1
	I inform informally in the hallway or some other place outside the office	16	2.5

**Table 2:** Further information on the ways of communicating bad news to cancer patients.

		*N*	%
How do you communicate the bad news to patients with cancer? (multiple choice)	With clear, understandable language, avoiding obscure medical terminology	537	84.8
	I explain in detail	246	38.9
	I give hope even if it doesn’t exist	169	26.7
	I create a relationship of trust	342	54.0
	I explain in detail and with special medical terminology	23	3.6
	I put myself in the patient’s shoes	182	28.8
	Clarify doubts	334	52.8
When you announce the bad news to patients with cancer, are you always telling the truth about diagnosis, prognosis and treatment?	I avoid telling the truth	8	1.3
	I’m saying it all at once	39	6.2
	Give (information) in a careful manner, according to the patient’s requirements and/or of the family	586	92.6
To whom are you telling the truth?	Only to the patient	11	1.7
	Only to the family	27	4.3
	To the patient and his/her partner, at the same time	60	9.5
	Preferably, first to the patient, then to the family	225	35.5
	Preferably, first to the family and then to the patient	310	49.0

**Table 3:** Interaction, feelings and fears during the communication of bad news to cancer patients.

When the oncology patient speaks and/or asks a question, you…: (multiple choice)	I listen carefully and without interrupting the patient	427	67.5
	I listen to the patient interrupting whenever there is something to add	139	22.0
	I don’t let the patient talk too much and I say things as they are	2	0.3
	I always take the time to answer his questions	456	72.0
What do you investigate during the conversation with the oncology patient? (multiple choice)	What the patient knows about his health condition	500	79.0
	What the patient wants to know	475	75.0
	What worries the patient	503	79.5
	I only inform, without giving the patient space to talk	5	0.8
How do you feel when you announce the bad news to oncology patients? (multiple choice)	Sorrow–Compassion	314	49.6
	Pity	27	4.3
	Feeling of fulfilling duty	194	30.6
	Relieved	13	2.1
	Anxiety	61	9.6
	Fear	5	0.8
	It doesn’t affect me	13	2.1
	Other (Uncomfortable; Frustration; Anger; Mental Fatigue)	5	0.8
What fear do you have when you announce the bad news to oncology patients? (multiple choice)	Fear of being accused	34	5.4
	Fear of the end of the patient’s hope	385	60.8
	Fear of death and the disease itself	109	17.2
	Fear of my own emotional reactions	94	14.8
	Fear of the patient’s reactions	341	53.9
	I have no fear	40	6.3
	Other (lack of information)	6	0.9

**Table 4:** Correlations between breaking bad news’ related variables and gender, hospital type, type of physicians and their experience.

		Male *N* = 400	Female *N* = 233	*p*-Value	Trainee *N* = 113	Specialist *N* = 520	*p*-Value	Public *N* = 365	Private *N* = 520	Anti-Cancer *N* = 75	*p*-Value	Experience *N* = 561	Education *N* = 72	*p*-Value
Delivering bad news	Verbal and emotional	316 (79.0)	207 (88.8)	**0.002**	91 (80.5)	432 (83.1)	0.497	294 (80.5)	167 (86.5)	62 (82.7)	0.208	453 (80.7)	70 (97.2)	**<0.0005**
	Verbal communication only	84 (21.0)	26 (11.2)		22 (19.5)	88 (16.9)		71 (19.5)	13 (17.3)	26 (13.5)		108 (19.3)	2 (2.8)	
Ability to deliver bad news	Poor–Acceptable	83 (20.8)	78 (33.5)	**0.001**	49 (43.4)	112 (21.5)	**<0.0005**	111 (30.4)	32 (16.6)	18 (24.0)	**0.002**	158 (28.2)	3 (4.2)	**<0.0005**
	Good	196 (49.0)	106 (45.5)		46 (40.7)	256 (49.2)		164 (44.9)	108 (49.2)	30 (40.0)		269 (48.0)	33 (45,8)	
	Very good	121 (30.3)	49 (21.0)		18 (15.9)	152 (29.2)		90 (24.7)	53 (27.5)	27 (36.0)		13 (23.9)	36 (50,0)	
Area of delivering bad news	Private and comfortable place	202 (50.5)	117 (50.2)	0.843	48 (42,5)	271 (52.1)	**<0.0005**	163 (44.7)	125 (64.8)	31 (41.3)	**<0.0005**	270 (48.1)	49 (68.1)	**0** **.** **006**
	Any available office	189 (47.3)	109 (46.8)		56 (49.6)	242 (46.5)		190 (52.1)	66 (34.2)	42 (56.0)		276 (49.2)	22 (30.6)	
	Hospital corridor	9 (2.3)	7 (3)		9 (8.0)	7 (1.3)		12 (3.3)	2 (1.0)	2 (2.7)		15 (2.7)	1 (1.4)	
How do you feel when you deliver bad news to patients with cancer?	Fulfilling my duty	151 (37.8)	43 (18.5)	**<0.0005**	32 (28.3)	162 (31.2)	0.080	112 (30.7)	59 (30.6)	23 (30.7)	0.587	167 (30.0)	27 (37.5)	0.788
	Relief	11 (2.8)	2 (0.9)		2 (1.8)	11 (2.1)		9 (2.5)	3 (1.6)	1 (1.3)		1 (2.0)	2 (2.8)	
	Anxiety	17 (4.3)	37 (15.9)		13 (11.5)	41 (7.9)		35 (9.6)	12 (6.2)	7 (9.3)		53 (9.4)	8 (11.1)	
	Sad	174 (43.5)	124 (53.4)		46 (40.7)	252 (48.5)		167 (45.8)	100 (51.8)	31 (41.3)		283 (50.4)	31 (43.1)	
	Pity	13 (3.3)	14 (6.0)		10 (8.8)	17 (3.3)		16 (4.4)	8 (4.1)	3 (4.0)		24 (4.3)	3 (4.2)	
	Fear	3 (0.8)	2 (0.9)		2 (1.8)	3 (0.6)		2 (0.5)	1 (0.5)	2 (2.7)		5 (0.9)	0 (0)	
What fears do you have when you deliver bad news to patients with cancer?	Blame the physician	20 (5)	14 (6)	0.588	6 (5.3)	28 (5.4)	1	14 (3.8)	12 (6.2)	8 (10.7)	0.100	31 (5.5)	3 (4.2)	0.787
	End of the patient’s hope	239 (59.8)	146 (62.7)	0.500	60 (53.1)	325 (62.5)	0.071	217 (59.5)	117 (60.6)	51 (68.0)	0.384	346 (61.7)	39 (54.2)	0.249
	Death and illness	64 (16.0)	45 (19.3)	0.326	18 (15.9)	91 (17.5)	0.784	59 (16.2)	35 (18.1)	15 (20.0)	0.669	97 (17.3)	12 (16.7)	1
	Emotional reaction of physician	48 (12)	46 (19.7)	**0.011**	11 (9.7)	83 (16.0)	0.108	54 (14.8)	30 (15.5)	10 (13.3)	0.900	48 (12.0)	46 (19.7)	0.601
	Patient reaction	198 (49.5)	143 (61.4)	**0.004**	68 (60.2)	273 (52.5)	0.146	200 (54.8)	102 (52.8)	39 (52.0)	0.856	305 (54.4)	36 (50.0)	0.531
	I am not afraid	34 (8.5)	6 (2.6)	**0.003**	6 (5.3)	34 (6.5)	0.624	30 (8.2)	9 (4.7)	1 (1.3)	0.088	31 (5.5)	9 (12.5)	0.065
How did you learn to deliver bad news?	By trying and learning	195 (48.8)	112 (48.1)	0.220	61 (54.0)	246 (47.3)	0.068	179 (49.0)	92 (47.7)	36 (48.0)	0.197	-	-	-
	Special training	39 (9.8)	33 (14.2)		8 (7.1)	64 (12.3)		35 (9.6)	30 (15.5)	7 (9.3)		-	-	
	During my studies	83 (20.8)	44 (18.9)		16 (14.2)	111 (21.3)		67 (18.4)	44 (22.8)	16 (21.3)		-	-	
	Meetings with experts	78 (19.5)	44 (18.9)		28 (24.8)	94 (18.1)		81 (22.2)	26 (13.5)	15 (20.0)		-	-	
	Other	5 (1.3)	0 (0)		5 (1.3)	0 (0)		3 (0.8)	1 (0.5)	1 (1.3)				

**Table 5:** Multivariable analysis on the ability to communicate bad news.

	Reference Category	OR	95%CI		*p*-Value
age (years)	---	1.03	1	1.05	0.018
gender (men)	women	1.63	1.10	2.42	0.015
specialty (specialized)	trainee	1.49	0.83	2.68	0.183
knowledge acquisition (training)	empirically	9.34	2.86	30.55	<0.0005
residence (outside Athens)	Athens	2.27	1.37	3.77	0.002
hospital					0.012
oncology hospital	public	1.90	1.02	3.54	0.043
private hospital		1.81	1.13	2.90	0.014

## Data Availability

Data sharing is not applicable to this article. The data are not publicly available due to restrictions. They contain information that could compromise the privacy of the participants.
